# Autonomic Nervous System Neuroanatomical Alterations Could Provoke and Maintain Gastrointestinal Dysbiosis in Autism Spectrum Disorder (ASD): A Novel Microbiome–Host Interaction Mechanistic Hypothesis

**DOI:** 10.3390/nu14010065

**Published:** 2021-12-24

**Authors:** Athanasios Beopoulos, Manuel Gea, Alessio Fasano, François Iris

**Affiliations:** 1 Bio-Modeling Systems, Tour CIT, 3 Rue de l’Arrivée, 75015 Paris, France; thanos.beopoulos@bmsystems.org (A.B.); manuel.gea@bmsystems.org (M.G.); 2 Mucosal Immunology and Biology Research Center, Center for Celiac Research and Treatment, Division of Pediatric Gastroenterology and Nutrition, Massachusetts General Hospital for Children, Boston, MA 022114, USA; afasano@mgh.harvard.edu

**Keywords:** autism spectrum disorder (ASD), autonomic nervous system (ANS), microbiome, dysbiosis, brain–gut axis (BGA), gastrointestinal (GI) tract, neurodevelopment

## Abstract

Dysbiosis secondary to environmental factors, including dietary patterns, antibiotics use, pollution exposure, and other lifestyle factors, has been associated to many non-infective chronic inflammatory diseases. Autism spectrum disorder (ASD) is related to maternal inflammation, although there is no conclusive evidence that affected individuals suffer from systemic low-grade inflammation as in many psychological and psychiatric diseases. However, neuro-inflammation and neuro–immune abnormalities are observed within ASD-affected individuals. Rebalancing human gut microbiota to treat disease has been widely investigated with inconclusive and contradictory findings. These observations strongly suggest that the forms of dysbiosis encountered in ASD-affected individuals could also originate from autonomic nervous system (ANS) functioning abnormalities, a common neuro–anatomical alteration underlying ASD. According to this hypothesis, overactivation of the sympathetic branch of the ANS, due to the fact of an ASD-specific parasympathetic activity deficit, induces deregulation of the gut–brain axis, attenuating intestinal immune and osmotic homeostasis. This sets-up a dysbiotic state, that gives rise to immune and osmotic dysregulation, maintaining dysbiosis in a vicious cycle. Here, we explore the mechanisms whereby ANS imbalances could lead to alterations in intestinal microbiome–host interactions that may contribute to the severity of ASD by maintaining the brain–gut axis pathways in a dysregulated state.

## 1. Introduction

The clinical manifestation of autism spectrum disorder (ASD) is frequently associated with gastrointestinal (GI) dysbiosis that is unrelated to dietary habits and is often manifested by altered bowel habits and chronic abdominal pain [1,2]. GI-related symptoms appear to strongly correlate with ASD severity [3]. Most of the reported dysbiosis observed in the feces and/or oral cavity/saliva of ASD children present an enormous heterogeneity in terms of microbial populations affected as well as in terms of types of alterations [4]. The only consistent observations appear to be a significant decrease in overall bacterial diversity together with an increased presence of the Bacteroidetes phylum (Gram-negative, non-spore-forming, and anaerobic), leading to the imbalance of the Bacteroidetes/Firmicutes (Gram-positive, spore-forming, and obligate or facultative aerobes bacilli) ratio (B/F ratio).

Interestingly, this very same observation (increased B/F ratio) has been reported in studies addressing depressive patients [5,6] as well as in depressive-like rat models [7,8]. While an increased B/F ratio is commonly associated with inflammatory bowel disease, reduced bacterial diversity and decreased B/F ratio (the opposite of ASD) appears common to many conditions such as obesity, diabetes T2, aging, etc. In association with autism, the genera most frequently reported as overrepresented include the Clostridium genus [9,10,11,12,13,14,15,16]. The Clostridium genus is also overrepresented in mice under chronic stress as well as in rodents separated from their mothers [17,18]. ASD-associated changes in microbiota diversity and genus abundance are directly related to autistic symptoms while being largely independent of dietary habits [15].

Individuals with ASD show four times more psychiatric and systemic comorbidities than those without ASD. These comorbidities include anxiety, mood disorders, depressive disorders, sleep disorders, and gastrointestinal dysfunctions. The prevalence of gastrointestinal problems in children with ASD is reported to range from 9% to 70%, extending from mild gastroesophageal reflux to more severe symptoms, with chronic constipation being the most frequently occurring complication with an average reported frequency of 22% [19]. Anxiety disorders are common in individuals with ASD with an overall prevalence ranging between 42% and 79%, the most common being specific phobia, obsessive-compulsive disorder, and social anxiety disorder. Their prevalence and nature can differ according to age and ability level with ASD-affected youngsters showing higher anxiety levels compared to clinically referred children [20]. In addition, a person with ASD is four times more likely to experience a depression disorder than a neurotypical individual [21]. Sleep disorders, such as insomnia (56%), bedtime resistance (54%), parasomnia (53%), morning rise problems (45%), daytime sleepiness (31%), and sleep-disordered breathing (25%) are important characteristic of individuals with ASD, impacting their social interactions, daily life activities, and academic performance [22,23].

Although there is considerable research on rebalancing the human gut microbiota to treat disease, the results are largely inconclusive and contradictory. The use of different methodologies, high incidence of antibiotic use, special diets, and/or repetitive feeding behaviors further complicate the issue [24]. For instance, mice treated with broad-spectrum antibiotics that affect anaerobic bacteria in favor of yeast flora [25] showed behavioral abnormalities commonly associated with ASD [26]. In human patients treated accordingly, it was observed that it took at least 40 days from the start of treatment for the microbiota to return to a state close to that before treatment [27,28].

Systematic reviews of a wide range of dietary manipulations, such as nutrient supplementation or gluten and casein-free diets, have found little evidence of attenuating effects on ASD symptoms [29,30]. The same is true for microbiota transfer therapy (MTT) when used to treat gastrointestinal problems commonly associated with ASD. Numerous studies of MTT have shown some improvements, albeit of short duration [31]. However, there is one recent open-label trial where MTT is reported to result in an 80% reduction in gastrointestinal symptoms including significant improvement in constipation and abdominal pain [32,33] with prolonged duration [34]. Hence, while MTT shows promises, in its current form, the results are far from generally maintainable without renewed intervention.

Oxytocin (OT), the neuropeptide of hypothalamic origin involved in the regulation of social behavior, has been suggested as a therapeutic approach for a number of psychiatric disorders characterized by social deficits, such as ASD, and several clinical studies have reported marginal benefits from intranasal oxytocin treatments of patients with ASD [35,36]. However, OT has several disadvantages as a clinical treatment because of its rapid metabolism, poor brain penetration, and activity at vasopressin receptors [37]. Therefore, continuous administration of intranasal OT alone for the treatment of social symptoms of high-functioning ASD in adults at current doses and duration cannot be recommended [38,39].

Nevertheless, although alterations and dysbiosis of the gut microbiota, accompanied by comorbidities, such as gastrointestinal problems and sleep disturbances, are features of the clinical manifestation of ASD, they are not limited to autism. They are also commonly observed in patients with psychiatric disorders, such as schizophrenia, major depressive disorder, and bipolar disorder [40], although frequently in combination with a chronic state of low-grade inflammation [41,42,43]. In contrast, even if neuroinflammation is most likely involved in ASD pathogenesis, the role of the immune system and inflammation in the development and persistence of the neurological and behavioral abnormalities characteristic to ASD remain inconclusive [44]. Indeed, while numerous new lines of evidence suggest that neuroinflammation is a common denominator in ASD, it remains virtually impossible to determine whether or not this could be a cause rather than a consequence of the numerous neurological and hormonal dysregulations resulting from the pathology [45].

Furthermore, there are three questions that remain seldom, if at all, addressed:

- Why is dysbiosis so prevalent in ASD?
- How does dysbiosis arise in the first place?
- How is it maintained?

## 2. Materials and Methods

The authors conducted an in-depth systematic review using a systems biology approach to integrate the complex mechanisms of the gut–brain axis to decipher the origins of dysbiosis encountered in individuals with ASD. The analytical procedure implemented (CADI™: computer-assisted deductive integration) associates algorithmics and heuristics. The logic behind this model-building approach does not assume functional linearity within biological systems, and the components of a model do not incorporate solely what is known. Indeed, since this approach relies upon strict and systematic implementation of the negative selection of hypotheses, models arising from this procedure contain elements that have never been described but cannot be refuted by current knowledge and/or available biological data, thereby generating novel understanding. This model-building approach has proven its efficacy in a number of biological research domains including the discovery of hitherto unsuspected biological mechanisms, pathways, and interactions directly associated with phenotypic transitions in vivo (be they pathological or developmental) [46,47,48,49,50,51,52,53,54]. CADI™ modeling has led to discoveries and patents in the fields of infectious diseases, oncology, neurology, psychiatry, dermatology, immunology, metabolic disorders, innovative bioprocesses for industrial biotech, and the creation of new companies exploiting these patents. CADI™ models describe the biological phenomena involved in pathological states and provide novel mechanistic integrations to explain the cause of certain diseases, identify and select predictive biomarkers, and offer new combinations of molecules and new therapeutic strategies. Further information on the CADI™ method can be found in [55].

## 3. Results and Discussion

### 3.1. A Novel “Intestinal Microbiome–Host Interaction” Hypothesis

Taken together, the above observations strongly suggest that the forms of dysbioses encountered in the context of ASD are much more complex than initially assumed and may be multifactorial involving mechanisms that are not mutually exclusive. Here, we present that beside the common causes of dysbiosis described for other chronic conditions, an attenuation of autonomically mediated stimulation [56] of exosomal secretion [57] by the colonic mucosa [58,59], probably together with decreased lectins secretion [60], may be at play in ASD. The consequence would be a decrease in the secretion of bactericidal peptides and metabolites from Paneth cells, goblet cells, and intestinal mucosal surface phagocytes, such as macrophages, which coexist with microbial communities [61]. This would lead to the loss of commensal microbial populations and the simultaneous deregulation of luminal ionic and water homeostasis, resulting in the highly heterogeneous and polymorphic dysbioses with unusually high incidences observed in ASD [26]. If not attenuated, these mechanisms would also prevent the establishment of commensal bacteria introduced by MTT, which would explain the generally transient nature of the improvements achieved by this approach.

However, for such events to become plausible, autonomic dysfunction in ASD-affected individuals would appear to be a prerequisite.

### 3.2. Autonomic Nervous System Dysfunction in Association with ASD

A common symptom in patients with ASD is the abnormal functioning of the autonomic nervous system (ANS) and specifically of the sympathetic branch of the ANS, which appears to be overactivated, primarily due to the fact of a deficit in parasympathetic activity [62,63,64,65]. This results in an autonomic imbalance, evidenced by a faster and less variable heart rate, respiratory dysrhythmia, a state of chronic sensory hyperexcitation, and increased tonic electrodermal activity. Indeed, heart rate variability in neurotypical and ASD individuals before, during, and after sleep provides characteristic autonomic distinctions in ASD adults. Specifically, during wakefulness, ASD adults had a lower high-frequency normalized spectral heart rate (HFnu), whereas during REM sleep, they had a higher low frequency/high frequency (LF/HF) ratio than children, regardless of clinical status [66,67].

### 3.3. The Autonomic Nervous System and Intestinal Immune Homeostasis

The ANS not only directly influences intestinal epithelial stem cell proliferation [68] but also governs intestinal peptides production which, in return, affects microbiota diversity (passive selective effects). The main effectors of intestinal innate immunity are Paneth and Goblet cells, located in the Lieberkühn crypts of the small intestine and scattered in the intestinal villi, respectively. Paneth cells are responsible for the production, storage, and secretion of several antimicrobial peptides (AMPs) such as secretory phospholipase A2 [69], lysozymes, regenerating islet-derived protein 3γ (RegIII-γ), and α-defensins [69,70,71].

Following parasympathetic cholinergic stimulation [70], Paneth cell degranulation is mediated either through KCa3.1 calcium-activated potassium channels [72] or through muscarinic receptors [73]. Activation of the latter results in calcium-dependent intracellular signal transduction through G-protein-coupled receptors [74] with both mechanisms dynamically increasing cytosolic calcium and subsequent granule secretion [75]. The antimicrobial-rich granules discharged are accumulated in the goblet cell mucus layer of the gastrointestinal tract, similar to sIgA [76,77], enforcing the mucosal barrier by preventing bacterial attachment and invasion [78]. They concurrently shape the composition of the indigenous microbiota [79,80] and protect the host from enteric pathogen infections [81,82,83].

Attenuation of the parasympathetic (cholinergic) inputs driving these antimicrobial activities weakens the host’s ability to retain commensals within the gut lumen and to prevent bacterial translocation [70]. These, in turn, affect intestinal metabolism, leading to constitutively high metabolic stress and the generation of reactive oxygen species (ROS) which for intestinal bacteria is equivalent to oxygen supply, thereby favoring aerobic/facultative anaerobic taxa over anaerobic/facultative aerobic taxa. This is likely to result in a vicious circle, one of the immediate effects of which will be to limit the generally observed medium-term efficacy of MTT by drastically impeding colonization by exogenously introduced so-called “beneficial microbial species” [84].

### 3.4. The ANS and the Neuronal Control of the Intestine

The gut is controlled by the autonomic nervous system, composed of the sympathetic, parasympathetic, and enteric nervous systems and by primary sensory afferents that communicate with immune cells for the release of neurotransmitters that regulate their activities. These interactions occur at several levels including the gut, central nervous system (CNS), and lymphoid organs [85]. The GI tract is densely innervated by the ENS, which comprises a network of 200–600 million neurons. It is segregated in the submucosa as ganglionic plexi forming the submucosal plexus, and within the longitudinal and circular muscle layers of the intestine, forming the myenteric plexus [86]. Parasympathetic stimulation results in increased activity of the whole ENS. Cranial parasympathetic nerve fibers innervate the proximal half of the nervous system through the vagal nerve, while sacral parasympathetic nerves innervate the distal half. These provide a rich neural input to the sigmoid colon, rectum, and anus and play an important role in the control of defecation.

Sympathetic fibers are rooted in the sympathetic ganglia of the T-5 to L-2 vertebraes and mostly end in the enteric plexuses, although some nerves terminate in the mucosa itself. Stimulation of enteric nerves by the sympathetic system has an inhibitory effect on GI activity. This is mainly accomplished through inhibition of enteric plexus actions and secondarily through the direct effect of secreted norepinephrine.

The intestine is also innervated by sensory afferent fibers. Their cell bodies are located either in the enteric plexuses or in the spinal cord. They are able to send information about irritation and overdistension, while also scanning the gut for chemical signals. About 80% of the vagus nerve fibers are afferent and their signals are processed by the medulla [87].

Besides autonomously influencing the physiology and function of the GI tract, the ENS can also communicate in a bidirectional manner with the CNS by both vagal parasympathetic and sympathetic pathways. Vagal afferent signaling from the ENS is mediated by intraganglionic laminar terminals, while signaling from the circular muscle layers and mucosa are facilitated by intramuscular networks and mucosal varicose nerve endings. All of these networks are composed of distinct neuronal populations identified by their function and morphology. They include enteric interneurons, intrinsic sensory neurons, and motor neurons (muscle, secretomotor, and vasodilator) that cooperate in the regulation of key functions of the gastrointestinal tract such as intestinal muscle activity, gastric peristalsis, as well as secretomotor and vasomotor activity [86]. The ENS, being located in the intestinal wall, is protected from the contents of the lumen by the epithelial barrier, the mucosal layer, and through ion and fluid secretion [88]. These barriers separate, to some extent, the ENS from the microbiota, as the GI tract is the most colonized space in the body with bacterial concentrations ranging from 10^1^ to 10^3^ cells per gram in the upper intestine to 10^11^–10^12^ per gram in the colon [89,90].

The afferent vagal neurons of the parasympathetic system transmit information to the CNS about the state of the gut. However, the gut has an additional network of afferent neurons and cell bodies within the enteric plexus which, instead of transmitting the information to the CNS, form part of a local and independent enteric control system [91]. Moreover, neuroendocrine cells dispersed in the epithelial monolayer are in close contact with the mucosal plexus and release their transmitters in response to stimuli. These are able to act in a paracrine manner on nearby epithelial cells and in a systemic manner through the activation of specific ENS receptors. [92].

### 3.5. The Brain–Gut Axis: Microbiome–Host Interactions and Immuno–Modulation

**Figure 1:**
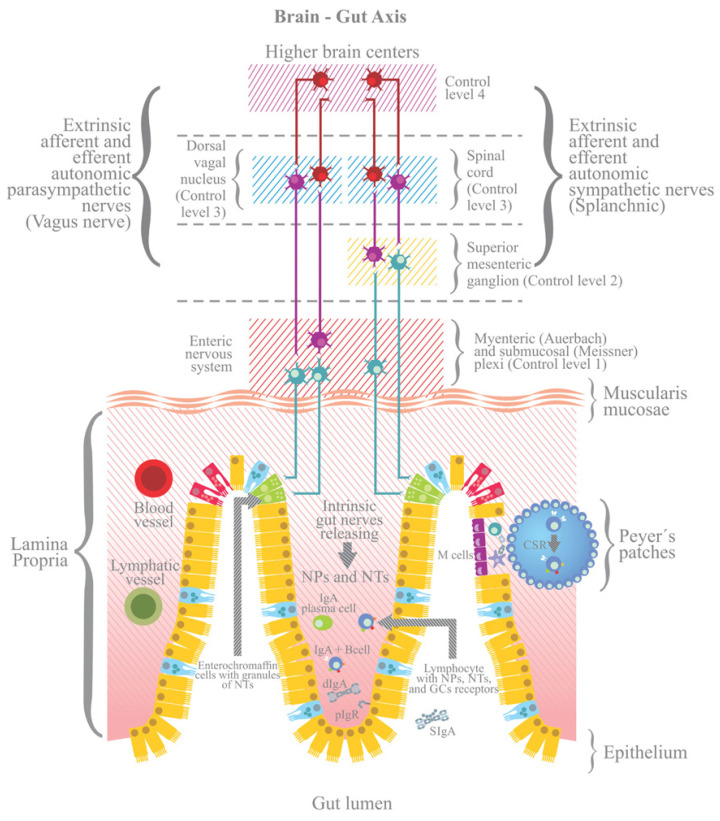
The ENS is connected to the CNS via the sympathetic and parasympathetic pathways, forming the brain–gut axis (BGA) [95]. Four levels for the control of the BGA are shown: activation of the ENS, including afferent and efferent intrinsic intestinal nerves (afferent nerves send signals from the periphery to the brain; efferent nerves from the brain to the periphery); and extrinsic innervations, whether sympathetic (splanchnic) or parasympathetic (vagus nerve) influences the generation of dIgA and/or the pIgR-mediated trancytosis. NTs, neurotransmitters; NPs, neuropeptides; GCs, glucocorticoids. Epithelial cell types: blue, enterochromaffin cells; green, neuroendocrine cells; red, Paneth cells; yellow, enterocytes.

The brain–gut axis (BGA) is formed by the sympathetic and parasympathetic pathways connecting the ENS to the CNS (Figure 1). Its responses are mediated by a complex network of mechanisms involving the hypothalamic–pituitary–adrenal axis, the sympathetic–parasympathetic–ENS axis and the brain–gut axis with the secretion of hormones, neurotransmitters and immune mediators that shape the humoral and cellular responses of the gut immune system [93]. For instance, enterochromaffin cells sense and transmit information from the gut to the central nervous system through the release of serotonin on primary afferent nerve fibers expressing 5-HT3 receptors that extend into the gut villi [94].

The gut is an important source of bioactive peptides with over 100 peptides existing in mammals, many of which are involved in gut–brain communication. In addition to their direct effects on peripheral and brain tissues, intestinal peptides may also influence enteric neurons. [96]. Since their concentrations are modulated by signals emitted from the resident microbiota, they vary according to its composition [97]. The signaling of gut peptides (GLP1/2, CCK, ghrelin, PYY, NPY, galanin, etc.) is therefore an important modulator of pathophysiological processes related to brain disorders such as anxiety, depression, and autism.

Furthermore, microorganisms are able to synthesize a large number of metabolites which have beneficial or detrimental properties for human health. The control of gut motility and/or the gut–brain axis is mediated by the interaction between gut microbiota and the ENS. Gut microbiota produce bioactive molecules that act on enteric neurons to influence GI motility, and, by impacting intrinsic primary afferent neurons (IPAN; [98,99]), modify the “gut–brain axis” signaling [98].

Nevertheless, the biochemical nature of the bacterial molecules involved in ENS is particularly broad, as they include a large number of nitrogenous molecules, such as oligopeptides and amino acids, with their derivatives. These molecules influence both the biosynthesis and regulation of enteric neurotransmitters, such as serotonin which is involved in the control of GI motility. As bacterial signaling peptides are N-formylated [100], their exact involvement in the control of host metabolism is still under investigation. Studies have shown that they can be detected by G-protein-coupled formyl peptide chemosensing receptors [101]. Although no direct link to their involvement in ENS is described, N-formyl-methionyl-leucyl-phenylalanine, derived from Gram-negative bacteria, has been shown to release NO from nerve cells in chicken embryos [102].

Another example is oxytocin which, apart from plasma, is detected in almost all segments of the GI tract [103]. Oxytocin is distributed in plasma after feeding [104], while it is also expressed in the myenteric and submucosal ganglia, suggesting its importance for gastrointestinal sensitivity and motility [105]. Oxytocin has analgesic effects [106], and its plasma levels are decreased in patients suffering from dyspepsia and irritable bowel syndrome [88], conditions characterized by abdominal pain and discomfort [107]. Furthermore, lower plasma levels of oxytocin are measured in children suffering from recurrent abdominal pain [108]. Transcripts for oxytocin and oxytocin receptors are strongly expressed in the intestines of adult mice and rats and in the precursors of enteric neurons in rat fetuses. Expression of enteric oxytocin and oxytocin receptors continues into adulthood but is developmentally regulated, peaking at postnatal day 7. In adults, approximately 1% of myenteric neurons express oxytocin, while 71% of myenteric plexus neurons, including primary submucosal and intrinsic afferent neurons, express oxytocin receptors. These receptors are also present in the nodose ganglia and, thus, oxytocin signaling could also influence extrinsic primary afferent neurons [109].

In the animal model of chronic colitis, anxiety-related behavior is vagally (parasympathetically) mediated, since it is absent in previously vagotomised animals. In mice, gut microbiota depletion in early adolescence affects oxytocin signaling, reducing hypothalamic oxytocin and vasopressin levels in stressed animals [110,111]. In parallel, the normalization of oxytocin levels in stressed mice reduces anxiety-like behavior and corrects cognitive deficits [110].

Thus, gut–brain communication transforms sensory information from the GI tract [94] into neural, immunological, and hormonal signals that are then interpreted independently or cooperatively by the CNS.

### 3.6. The ANS and the Control of Intestinal Immune Homeostasis

The ANS, in addition to regulating intestinal motility, absorption, and secretion, plays a key role in modulating immune function and intestinal homeostasis through its communication with the immune system [112]. Deregulation at any level could therefore lead to gastrointestinal disorders related to food and/or bacterial intolerance, indicating that its contribution to immune function should not be underestimated.

Indeed, pro- and anti-inflammatory interleukins (ILs), endocrine hormones, and neurotransmitters control intestinal homeostasis by regulating intestinal secretions, thus affecting intestinal immunity and permeability. Microorganism colonization of the gut may be essential for several functions of gastrointestinal physiology [113,114] but also for the maturation of the mucosal immune system [115]. For example, in a study using germ-free mice to investigate the electrophysiological properties of neurons in the myenteric plexus of the ENS, it was shown that the commensal microbiota is necessary for normal excitability of intestinal sensory neurons [99]. Further studies on this topic confirmed that germ-free mice have fewer excitable intrinsic primary afferent neurons [116], which may be enhanced by exposure to polysaccharide A [117]. Another study using germ-free mice confirmed the previous findings and showed that the microbiome is critical for both intrinsic and extrinsic neural function and gut–brain signaling [98]. Studies looking at the influence of the microbiota on postnatal development of the ENS found that germ-free mice had lower nerve density and fewer neuronal cell bodies in the myenteric ganglia, while the small intestine was found to have an increased proportion of inhibitory nitroergic neurons [118]. These results support the hypothesis that early exposure to luminal microorganisms is crucial for the postnatal development of the ENS.

Nerve fibers of the sympathetic nervous system (SNS), which has both pro- and anti-inflammatory functions, penetrate the enteric plexuses and innervate the mucosa and gut-associated lymphoid tissue (GALT). Norepinephrine, adenosine, and other neurotransmitters may have diverse or opposing effects depending on their concentration, the expression levels of adrenoreceptors, their binding affinity to receptor subtypes, the presence of co-transmitters, and the timing of SNS activity relative to the development of inflammation [119]. The relationship between stress and intestinal inflammation provides indirect evidence of this interaction, as stress can affect multiple functions of the mucosal barrier including permeability, microbial composition, IgA and mucin secretion [120].

This originates from the close association of the immune system and the nervous system. The latter reacts to environmental changes by releasing neurotransmitters and neuropeptides that by binding to the G-coupled receptors of the former, can act as immune modulators or directly modulate the profile of immune cells. This is also the case with the ENS, as its location by innervating the lamina propria and being in close contact with epithelial and neuroendocrine cells makes it a main sensor and modulator of immediate non-specific inflammatory responses. The ENS works in synergy with the immune system to orchestrate the body’s response to pathogens. For example, enteric glial cells regulate intestinal barrier function by releasing S-nitrosoglutathione, which then upregulates tight junction protein expression in epithelial cells. In contrast, adult transgenic mice, in which enteric glial cells are deleted, develop fulminant jejunoileitis due to the increased intestinal permeability [121].

To address the role of stress as an immune modulator, experimental assays have demonstrated how stress modulates secretory immunoglobulin A (SIgA) production [122,123] and polymeric immunoglobulin receptor (pIgR) expression [124]. The latter transports by transcytosis the immunoglobin–pIgR complexes (i.e., dIgA–pIgR and pIgA–pIgR) through the intestinal epithelial cells. SIgA, which is the most abundant class of antibodies in the intestinal lumen of humans, is the first line of defense in protecting the intestinal epithelium from enteric pathogens and toxins. SIgA facilitates the removal of antigens and pathogenic microorganisms from the intestinal lumen by trapping them in the mucus, blocking their access to epithelial receptors and facilitating their removal by peristaltic and mucociliary activities. It further influences the composition of the gut microbiota through Fab-dependent and Fab-independent mechanisms, eliminates bacterial virulence factors, promotes antigen back-transport by the gut epithelium to dendritic cell (DC) subsets of gut-associated lymphoid tissue, and downregulates inflammatory responses associated with allergenic antigens and pathogenic bacteria [125].

### 3.7. Persistent Dysbiosis in the Absence of Chronic Low-Grade Inflammation

**Figure 2:**
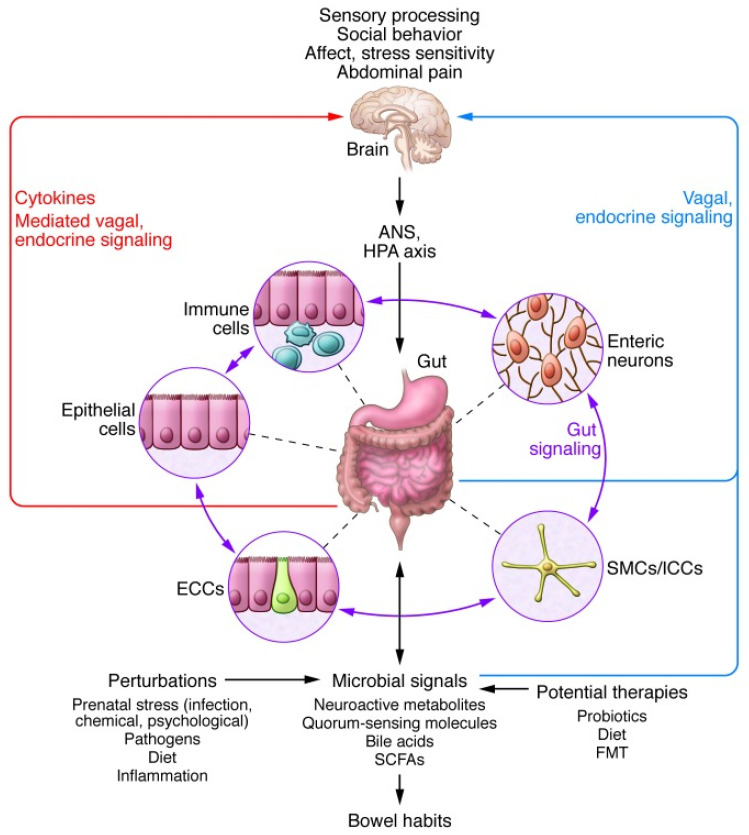
Bidirectional interactions within the gut microbiota/brain axis [126]. A network of specialized target/transducer cells in the gut wall functions as an interface between the microbiota and the host lumen. In response to external and bodily demands, the brain modulates these specialized cells within this network via the branches of the ANS (sympathetic and parasympathetic/vagal efferents) and the HPA axis. Such modulation can be transient, such as in response to transient perturbations, or long lasting such as in response to chronically altered brain output. The microbiota are in constant bidirectional communication with this interface via multiple microbial signaling pathways, and this communication is modulated in response to perturbations of the microbiota or the brain. The integrated output of the gut microbial–brain interface is transmitted back to the brain via multiple afferent signaling pathways including the endocrine (metabolites, cytokines, and microbial signaling molecules) and neurocrine (vagal and spinal afferents). While acute alterations in this interoceptive feedback can result in transient functional brain changes (GI infections), chronic alterations are associated with neuroplastic brain changes. Potential therapies aim to normalize altered microbiota signaling to the ENS and central nervous system. ECC, enterochromaffin cells; FMT, fecal microbial transplant; ICC, interstitial cell of Cajal; SCFA, short-chain fatty acid; SMC, smooth muscle cell.

The autonomic nervous system regulates inflammatory responses regionally via the innervation of lymphoid organs. Vagal efferents from the dorsal motor nucleus of the vagus modulate intestinal immune functions indirectly via inputs to the enteric nervous system (Figure 2). Studies in mice show that the sympathetic output from the celiac ganglia to the spleen activates the cholinergic anti-inflammatory pathway, eliciting a potent anti-inflammatory response. Hence, noradrenaline released from the SNS suppresses inflammatory cytokine production by macrophages and DCs and halts the chemotactic response of the latter to CCR7 ligands [85].

Vagal and dorsal root ganglion afferents innervating the gut express TLRs and receptors for inflammatory cytokines, such as IL-1β and tumor necrosis factor (TNF)-α, and relay inflammatory and immune signals to the CNS. Vagal afferents relay these signals to the nucleus of the solitary tract (NTS) and dorsal root ganglion afferents to lamina I of the spinal cord. Upon activation, sensory afferents may regulate local vascular and immune responses through antidromic signaling mediated by the release of substance P (SP), calcitonin gene-related peptide (CGRP), and other neuropeptides; circulating pro-inflammatory cytokines, such as IL-1 and IL-6, may reach receptors in endothelial cells of the blood–brain barrier (BBB) leading to synthesis of prostaglandin E2 (PgE2), which acts as a paracrine signal that regulates the function of hypothalamic nuclei such as the medial preoptic area [127] and the paraventricular nucleus (PVN). PgE2 also activates vagal and dorsal root ganglion afferents, while cytokines may also gain access to the CNS through their transport across the BBB or interactions with receptors in the circumventricular organs. Thus, the mucosal immune system can be modulated at the level of the central, autonomic, peripheral, and enteric nervous systems [112]. The peripheral and enteric nervous systems modulate responses locally via neuropeptide release such as calcitonin gene-elated peptide 5 (CGRP5), corticotropin-releasing hormone (CRH), and anti-melanocyte-stimulating hormone (AMSH). All these enhance the inflammatory response, while substance P (SP) triggers the release of serotonin and histamine through degranulation of mast cells and neuroendocrine cells, which further amplifies the inflammatory response, or through the release of vasoactive intestinal peptide (VIP) which inhibits the inflammatory reaction [128].

Indeed, while lipopolysaccharides (LPS) induce TNF-α production by enteric neurons through activation of the canonical ERK pathway and also in an AMP-activated protein kinase (AMPK)-dependent manner, ENS activation via electrical stimulation inhibits these pathways, decreasing TNF-α production and thereby down-modulating the inflammatory response induced by endotoxin [129]. Upon stimulation or in response to inflammatory mediators, such as IL-1 and TNF, sympathetic neurons also release noradrenaline and neuropeptide Y (NPY). While NPY inhibits natural killer cell activation [130], noradrenaline activates the splenic sympathetic anti-inflammatory pathway [131]. This response, resulting from noradrenaline release at the distal end of the splenic nerve, is mediated by activation of β2-adrenoceptors in a population of T cells that express choline acetyltransferase, indicating their potential for acetylcholine synthesis [132]. Activation of β2-adrenoceptor in these T cells triggers the synthesis and the release of acetylcholine [133], which, via α7-subunit containing acetylcholine receptors (α7nAChRs) expressed on macrophages and other immune cells, suppresses the release of TNF-α and pro-inflammatory cytokines [134].

Concomitantly, stimulation of the vagus (parasympathetic) nerve, composed of approximately 80% afferent and 20% efferent fibers, affects homeostatic regulation of visceral immune functions with anti-inflammatory effects [131,135]. Vagal afferent fiber stimulation of the adrenal glands results in activation of the hypothalamic–pituitary–adrenal (HPA) axis and release of cortisol, while efferent fiber stimulation releases acetylcholine (ACh) at the synaptic junction with macrophages [136]. Binding of acetylcholine (Ach) to α7nAChRs from these macrophages inhibits TNF-α release and pro-inflammatory cytokine production as described above [131,137]. Finally, vagal afferent fibers can stimulate the splenic sympathetic nerve, thereby further enhancing the splenic sympathetic anti-inflammatory pathway [138].

Hence, the ANS functioning abnormalities observed in association with autism (sympathetic over-activation on a background of parasympathetic activity deficits) are very likely to affect neuro–immune interactions, favoring anti-inflammatory enteric responses. This could, in turn, account for both the persistence of dysbiosis together with a lack of evidence for chronic low-grade inflammation in association with ASD, whereas most other psychiatric disorders associated with persistent dysbiosis show concurrently present chronic low-grade inflammation but no evidence of autonomic nervous system (ANS) functioning abnormalities.

### 3.8. ANS Functioning Abnormalities and Gastrointestinal Dysfunctions

As already stated above, GI problems in children with ASD can range from mild gastro-esophageal reflux to more severe symptoms such as chronic constipation, abdominal pain, and persistent diarrhea. The most common of these appears to be chronic constipation with a median prevalence of 22% [19].

The digestive tract responds to luminal contents through close integration of the interactions between enteroendocrine, neural, and tissue defense systems. The endocrine intestinal cells (EECs) contain glucagon-like peptides GLP-1, GLP-2, and peptide YY (PYY) in the distal small intestine. The GLP/PYY cells have luminal receptors for fats, carbohydrates, protein metabolites, and bile salts that regulate their responses [139,140]. They also receive input from enteric neurons. Activation of a vagovagal reflex and local nerve stimulation can both enhance hormone secretion from the GLP/PYY cells [141,142]. Many of the effects of hormones released by GLP/PYY cells are indirect, via activation of neurons. This is certainly the case with GLP-1- and PYY-mediated satiety. GLP-1 is inactivated by dipeptidyl peptidase-4 (DPPIV) to such an extent that it does not reach sites other than vagal nerve terminals in sufficient concentration to affect feeding behavior [143]. GLP-2 also promotes mucosal growth and repair, increases amino acid and fat absorption, enhances digestive enzymes’ activities, improves gut barrier function [144,145], and has anti-inflammatory effects [146]. Because of these effects, GLP-2 receptor agonists have potential in the treatment of short bowel syndrome and inflammatory bowel disease [147,148]. In contrast to GLP-1 and GLP-2, PYY consistently induces antisecretory/absorptive responses, reducing water and electrolyte secretion, primarily by acting to inhibit enteric secretomotor neurons, but also by acting on the enterocytes [149,150].

The same EECs can secrete hormones with different and, in some cases, even opposite effects, because different hormones, such as ghrelin and nesfatin, which induce hunger and satiety, respectively, are housed in distinct subcellular stores [151]. Similarly, GLP and PYY, which have opposing effects on fluid secretion and on glucose stimulation of insulin secretion, are stored separately in the same small intestinal EECs [152].

Over two volumes of blood pass through the mucosal epithelial surfaces each day, and any disruption in the regulation of fluid transport becomes life-threatening. This high flux is due in part to the fact that active uptake (against the chemical gradient) of sugars (monosaccharides) and amino acids occurs via cation-coupled transporters. Thus, when glucose is absorbed through the sodium/glucose-coupled transporter, it is internalized with a sodium ion and counter ions, which are mostly chloride ions. The absorption of 100 g of glucose is estimated to be equivalent to the absorption of 1.8 L of water [86]. Intestinal reflexes transport water and electrolytes from the interstitium of the lamina propria into the lumen through the activation of secretomotor neurons. Control over this process is exerted by blood volume and blood pressure detectors that alter the activity of two sympathetic pathways, the vasoconstrictor and secretomotor inhibitory pathways [86], so that fluid balance is maintained.

Glucose sensors are located on enteroendocrine cells [153] and stimulation of these cells releases GLP-2, as well as other hormones. GLP-2 receptors are located on non-cholinergic secretory neurons that are activated by this hormone [154,155]. Thus, activation of the enteric receptor for glucose by glucose or artificial sweeteners stimulates secretomotor neurons to return water and electrolytes to the lumen. Furthermore, neurons activated by GLP-2 increase glucose uptake through SGLT1 [155].

However, the fine control of water balance by local (ENS) and systemic (sympathetic) reflexes is deregulated when the lumen contains an excess of pathogens and/or their toxins, such as cholera toxin, rotaviruses, and pathogenic Escherichia coli, which are capable of activating enteric secretomotor neurons [156,157]. In mild cases, induced diarrhea expels pathogens and their toxic products. However, when high levels of pathogens or toxins are present in the gut, prolonged diarrhea can develop with potentially fatal consequences.

Hence, ANS functioning abnormalities, such as those observed in individuals with autism where the sympathetic branch of the ANS presents an over-activation on a background of parasympathetic activity deficits, creating an autonomic imbalance, will necessarily affect water and electrolyte movements across mucosal epithelial surfaces. All the more so in the presence of dysbiosis, which is now very likely to implement a vicious cycle, since transient osmotic perturbation causes long-term alteration to the gut microbiota [158], while long-term alteration to the gut microbiota promotes osmotic perturbations [159]. Whether the final effect will be chronic constipation (excessive absorption of water, see PYY above) or persistent diarrhea will then depend upon both the levels of autonomic imbalance and the nature of the concurrently present dysbiosis.

## 4. Conclusions

The novel host–microbiome interaction hypothesis embodied by this work can be summarized as follows:

- The neuro–anatomical alterations that underlie ASD provoke autonomic nervous system (ANS) functioning abnormalities characterized by an over-activation of the sympathetic branch of the ANS on a background of parasympathetic activity deficits;
- This induces deregulation of the gut–brain axis;
- This results in attenuation of the humoral and cellular components of the intestinal immune system, together with dysregulation of intestinal osmotic homeostasis and mucus production, with ensuing, persistent dysbiosis, which then sets-up a vicious cycle where immune and osmotic functioning abnormalities maintain a dysbiotic state which, in turn, maintains immune and osmotic dysregulation.

Taken globally, the mechanisms implemented in the context of this hypothesis can, for the first time, adequately explain:

- One of the possible origins of ASD-associated dysbiosis;
- Its persistence;
- Why improvements achieved by microbiota transfer therapy are generally transient;
- Why, in spite of persistent dysbiosis, ASD is not associated with systemic chronic low-grade inflammation (as opposed to neuroinflammation), while all other psychiatric disorders also associated with persistent dysbiosis are;
- The origins of the unusually high incidences of gastrointestinal dysfunctions;
- The high frequency of comorbidities such as sleep disorders, anxiety, and depression;
- Perhaps most importantly, the reasons for the very high heterogeneities in terms of forms and types of dysbioses as well as in frequencies of comorbidities associated with clinical ASD.

## References

[1] Coury D.L., Ashwood P., Fasano A., Fuchs G., Geraghty M., Kaul A., Mawe G., Patterson P., Jones N.E. (2012). Gastrointestinal conditions in children with autism spectrum disorder: Developing a research agenda. Pediatrics.

[2] Horvath K., Perman J.A. (2002). Autistic disorder and gastrointestinal disease. Curr. Opin. Pediatr..

[3] Adams J.B., Audhya T., McDonough-Means S., Rubin R.A., Quig D., Geis E., Gehn E., Loresto M., Mitchell J., Atwood S. (2011). Nutritional and metabolic status of children with autism vs. neurotypical children, and the association with autism severity. Nutr. Metab..

[4] Mangiola F., Ianiro G., Franceschi F., Fagiuoli S., Gasbarrini G., Gasbarrini A. (2016). Gut microbiota in autism and mood disorders. World J. Gastroenterol..

[5] Jiang H., Ling Z., Zhang Y., Mao H., Ma Z., Yin Y., Wang W., Tang W., Tan Z., Shi J. (2015). Altered fecal microbiota composition in patients with major depressive disorder. Brain Behav. Immun..

[6] Naseribafrouei A., Hestad K., Avershina E., Sekelja M., Linlokken A., Wilson R., Rudi K. (2014). Correlation between the human fecal microbiota and depression. Neurogastroenterol. Motil..

[7] Kelly J.R., Borre Y., O’Brian C., Patterson E., El Aidy S., Deane J., Kennedy P.J., Beers S., Scott K., Moloney G. (2016). Transferring the blues: Depression-associated gut microbiota induces neurobehavioural changes in the rat. J. Psychiatr. Res..

[8] Yu M., Jia H., Zhou C., Yang Y., Zhao Y., Yang M., Zou Z. (2017). Variations in gut microbiota and fecal metabolic phenotype associated with depression by 16S rRNA gene sequencing and LC/MS-based metabolomics. J. Pharm. Biomed. Anal..

[9] Finegold S.M., Molitoris D., Song Y., Liu C., Vaisanen M.L., Bolte E., McTeague M., Sandler R., Wexler H., Marlowe E.M. (2002). Gastrointestinal microflora studies in late-onset autism. Clin. Infect. Dis. Off. Publ. Infect. Dis. Soc. Am..

[10] Song Y., Liu C., Finegold S.M. (2004). Real-time PCR quantitation of clostridia in feces of autistic children. Appl. Environ. Microbiol..

[11] Parracho H.M., Bingham M.O., Gibson G.R., McCartney A.L. (2005). Differences between the gut microflora of children with autistic spectrum disorders and that of healthy children. J. Med. Microbiol..

[12] Finegold S.M., Dowd S.E., Gontcharova V., Liu C., Henley K.E., Wolcott R.D., Youn E., Summanen P.H., Granpeesheh D., Dixon D. (2010). Pyrosequencing study of fecal microflora of autistic and control children. Anaerobe.

[13] Wang L., Christophersen C.T., Sorich M.J., Gerber J.P., Angley M.T., Conlon M.A. (2013). Increased abundance of Sutterella spp. and Ruminococcus torques in feces of children with autism spectrum disorder. Mol. Autism.

[14] Adams J.B., Johansen L.J., Powell L.D., Quig D., Rubin R.A. (2011). Gastrointestinal flora and gastrointestinal status in children with autism–comparisons to typical children and correlation with autism severity. BMC Gastroenterol..

[15] Kang D.W., Park J.G., Ilhan Z.E., Wallstrom G., Labaer J., Adams J.B., Krajmalnik-Brown R. (2013). Reduced incidence of Prevotella and other fermenters in intestinal microflora of autistic children. PLoS ONE.

[16] Williams B.L., Hornig M., Parekh T., Lipkin W.I. (2012). Application of novel PCR-based methods for detection, quantitation, and phylogenetic characterization of Sutterella species in intestinal biopsy samples from children with autism and gastrointestinal disturbances. mBio.

[17] Bailey M.T., Dowd S.E., Galley J.D., Hufnagle A.R., Allen R.G., Lyte M. (2011). Exposure to a social stressor alters the structure of the intestinal microbiota: Implications for stressor-induced immunomodulation. Brain Behav. Immun..

[18] De Palma G., Blennerhassett P., Lu J., Deng Y., Park A.J., Green W., Denou E., Silva M.A., Santacruz A., Sanz Y. (2015). Microbiota and host determinants of behavioural phenotype in maternally separated mice. Nat. Commun..

[19] Tye C., Runicles A.K., Whitehouse A.J.O., Alvares G.A. (2018). Characterizing the Interplay Between Autism Spectrum Disorder and Comorbid Medical Conditions: An Integrative Review. Front. Psychiatry.

[20] Kent R.S., Kerns C.M., Renno P., Storch E.A., Kendall P.C., Wood J.J. (2017). Front-matter. Anxiety in Children and Adolescents with Autism Spectrum Disorder.

[21] Hudson C.C., Hall L., Harkness K.L. (2019). Prevalence of Depressive Disorders in Individuals with Autism Spectrum Disorder: A Meta-Analysis. J. Abnorm. Child Psychol..

[22] Klukowski M., Wasilewska J., Lebensztejn D. (2015). Sleep and gastrointestinal disturbances in autism spectrum disorder in children. Dev. Period. Med..

[23] Liu X., Hubbard J.A., Fabes R.A., Adam J.B. (2006). Sleep disturbances and correlates of children with autism spectrum disorders. Child Psychiatry Hum. Dev..

[24] Louis P. (2012). Does the human gut microbiota contribute to the etiology of autism spectrum disorders?. Dig. Dis. Sci..

[25] Samonis G., Gikas A., Anaissie E.J., Vrenzos G., Maraki S., Tselentis Y., Bodey G.P. (1993). Prospective evaluation of effects of broad-spectrum antibiotics on gastrointestinal yeast colonization of humans. Antimicrob. Agents Chemother..

[26] Sgritta M., Dooling S.W., Buffington S.A., Momin E.N., Francis M.B., Britton R.A., Costa-Mattioli M. (2019). Mechanisms Underlying Microbial-Mediated Changes in Social Behavior in Mouse Models of Autism Spectrum Disorder. Neuron.

[27] Perez-Cobas A.E., Gosalbes M.J., Friedrichs A., Knecht H., Artacho A., Eismann K., Otto W., Rojo D., Bargiela R., von Bergen M. (2013). Gut microbiota disturbance during antibiotic therapy: A multi-omic approach. Gut.

[28] Dethlefsen L., Huse S., Sogin M.L., Relman D.A. (2008). The pervasive effects of an antibiotic on the human gut microbiota, as revealed by deep 16S rRNA sequencing. PLoS Biol..

[29] Sathe N., Andrews J.C., McPheeters M.L., Warren Z.E. (2017). Nutritional and Dietary Interventions for Autism Spectrum Disorder: A Systematic Review. Pediatrics.

[30] Li Y.J., Li Y.M., Xiang D.X. (2018). Supplement intervention associated with nutritional deficiencies in autism spectrum disorders: A systematic review. Eur. J. Nutr..

[31] Kang D.W., Adams J.B., Vargason T., Santiago M., Hahn J., Krajmalnik-Brown R. (2020). Distinct Fecal and Plasma Metabolites in Children with Autism Spectrum Disorders and Their Modulation after Microbiota Transfer Therapy. mSphere.

[32] Kang D.W., Adams J.B., Gregory A.C., Borody T., Chittick L., Fasano A., Khoruts A., Geis E., Maldonado J., McDonough-Means S. (2017). Microbiota Transfer Therapy alters gut ecosystem and improves gastrointestinal and autism symptoms: An open-label study. Microbiome.

[33] Kang D.W., Adams J.B., Coleman D.M., Pollard E.L., Maldonado J., McDonough-Means S., Caporaso J.G., Krajmalnik-Brown R. (2019). Long-term benefit of Microbiota Transfer Therapy on autism symptoms and gut microbiota. Sci. Rep..

[34] Adams J.B., Borody T.J., Kang D.W., Khoruts A., Krajmalnik-Brown R., Sadowsky M.J. (2019). Microbiota transplant therapy and autism: Lessons for the clinic. Expert Rev. Gastroenterol. Hepatol..

[35] Parker K.J., Oztan O., Libove R.A., Sumiyoshi R.D., Jackson L.P., Karhson D.S., Summers J.E., Hinman K.E., Motonaga K.S., Phillips J.M. (2017). Intranasal oxytocin treatment for social deficits and biomarkers of response in children with autism. Proc. Natl. Acad. Sci. USA.

[36] Tachibana M., Kagitani-Shimono K., Mohri I., Yamamoto T., Sanefuji W., Nakamura A., Oishi M., Kimura T., Onaka T., Ozono K. (2013). Long-term administration of intranasal oxytocin is a safe and promising therapy for early adolescent boys with autism spectrum disorders. J. Child. Adolesc. Psychopharmacol..

[37] Moy S.S., Teng B.L., Nikolova V.D., Riddick N.V., Simpson C.D., Van Deusen A., Janzen W.P., Sassano M.F., Pedersen C.A., Jarstfer M.B. (2019). Prosocial effects of an oxytocin metabolite, but not synthetic oxytocin receptor agonists, in a mouse model of autism. Neuropharmacology.

[38] Yamasue H., Okada T., Munesue T., Kuroda M., Fujioka T., Uno Y., Matsumoto K., Kuwabara H., Mori D., Okamoto Y. (2018). Effect of intranasal oxytocin on the core social symptoms of autism spectrum disorder: A randomized clinical trial. Mol. Psychiatry.

[39] Kruppa J.A., Gossen A., Oberwelland Weiss E., Kohls G., Grossheinrich N., Cholemkery H., Freitag C.M., Karges W., Wolfle E., Sinzig J. (2019). Neural modulation of social reinforcement learning by intranasal oxytocin in male adults with high-functioning autism spectrum disorder: A randomized trial. Neuropsychopharmacol. Off. Publ. Am. Coll. Neuropsychopharmacol..

[40] Huang T.T., Lai J.B., Du Y.L., Xu Y., Ruan L.M., Hu S.H. (2019). Current Understanding of Gut Microbiota in Mood Disorders: An Update of Human Studies. Front. Genet..

[41] Rosenblat J.D., McIntyre R.S. (2017). Bipolar Disorder and Immune Dysfunction: Epidemiological Findings, Proposed Pathophysiology and Clinical Implications. Brain Sci..

[42] Rosenblat J.D., Brietzke E., Mansur R.B., Maruschak N.A., Lee Y., McIntyre R.S. (2015). Inflammation as a neurobiological substrate of cognitive impairment in bipolar disorder: Evidence, pathophysiology and treatment implications. J. Affect. Disord..

[43] Rosenblat J.D. (2019). Targeting the immune system in the treatment of bipolar disorder. Psychopharmacology.

[44] Dipasquale V., Cutrupi M.C., Colavita L., Manti S., Cuppari C., Salpietro C. (2017). Neuroinflammation in Autism Spectrum Disorders: Role of High Mobility Group Box 1 Protein. Int J. Mol. Cell Med..

[45] Eissa N., Sadeq A., Sasse A., Sadek B. (2020). Role of Neuroinflammation in Autism Spectrum Disorder and the Emergence of Brain Histaminergic System. Lessons Also for BPSD?. Front. Pharm..

[46] Gadal F., Bozic C., Pillot-Brochet C., Malinge S., Wagner S., Le Cam A., Buffat L., Crepin M., Iris F. (2003). Integrated transcriptome analysis of the cellular mechanisms associated with Ha-ras-dependent malignant transformation of the human breast epithelial MCF7 cell line. Nucleic Acids Res..

[47] Gadal F., Starzec A., Bozic C., Pillot-Brochet C., Malinge S., Ozanne V., Vicenzi J., Buffat L., Perret G., Iris F. (2005). Integrative analysis of gene expression patterns predicts specific modulations of defined cell functions by estrogen and tamoxifen in MCF7 breast cancer cells. J. Mol. Endocrinol..

[48] Iris F. (2012). Psychiatric systems medicine: Closer at hand than anticipated but not with the expected portrait. Pharmacopsychiatry.

[49] Iris F., Filiou M., Turck C.W. (2014). Differential Proteomics Analyses Reveal Anxiety-Associated Molecular and Cellular Mechanisms in Cingulate Cortex Synapses. Am. J. Psychiatry Neurosci..

[50] Iris F., Gea M., Lampe P.H., Santamaria P. (2009). [Production and implementation of predictive biological models]. Med. Sci. (Paris).

[51] Iris F.J.M., Gea M., Lampe P.-H., Querleux B. (2014). Heuristic Modelling Applied to Epidermal Homeostasis. Computational Biophysics of the Skin.

[52] Nussbaumer M., Asara J.M., Teplytska L., Murphy M.P., Logan A., Turck C.W., Filiou M.D. (2016). Selective Mitochondrial Targeting Exerts Anxiolytic Effects In Vivo. Neuropsychopharmacol. Off. Publ. Am. Coll. Neuropsychopharmacol..

[53] Pouillot F., Blois H., Iris F. (2010). Genetically engineered virulent phage banks in the detection and control of emergent pathogenic bacteria. Biosecur. Bioterror..

[54] Turck C.W., Iris F. (2011). Proteome-based pathway modelling of psychiatric disorders. Pharmacopsychiatry.

[55] Iris F., Beopoulos A., Gea M. (2018). How scientific literature analysis yields innovative therapeutic hypothesis through integrative iterations. Curr. Opin. Pharm..

[56] Furness J.B. (2016). Integrated Neural and Endocrine Control of Gastrointestinal Function. Adv. Exp. Med. Biol..

[57] Fleshner M., Crane C.R. (2017). Exosomes, DAMPs and miRNA: Features of Stress Physiology and Immune Homeostasis. Trends Immunol..

[58] Xu A.T., Lu J.T., Ran Z.H., Zheng Q. (2016). Exosome in intestinal mucosal immunity. J. Gastroenterol. Hepatol..

[59] Willemze R.A., Welting O., van Hamersveld P., Verseijden C., Nijhuis L.E., Hilbers F.W., Meijer S.L., Heesters B.A., Folgering J.H.A., Darwinkel H. (2019). Loss of intestinal sympathetic innervation elicits an innate immune driven colitis. Mol. Med..

[60] Pang X., Xiao X., Liu Y., Zhang R., Liu J., Liu Q., Wang P., Cheng G. (2016). Mosquito C-type lectins maintain gut microbiome homeostasis. Nat. Microbiol..

[61] Peck B.C.E., Shanahan M.T., Singh A.P., Sethupathy P. (2017). Gut Microbial Influences on the Mammalian Intestinal Stem Cell Niche. Stem Cells Int..

[62] Wang Y., Hensley M.K., Tasman A., Sears L., Casanova M.F., Sokhadze E.M. (2016). Heart Rate Variability and Skin Conductance During Repetitive TMS Course in Children with Autism. Appl. Psychophysiol. Biofeedback.

[63] Ming X., Patel R., Kang V., Chokroverty S., Julu P.O. (2016). Respiratory and autonomic dysfunction in children with autism spectrum disorders. Brain Dev..

[64] Kong X., Liu J., Liu K., Koh M., Tian R., Hobbie C., Fong M., Chen Q., Zhao M., Budjan C. (2021). Altered Autonomic Functions and Gut Microbiome in Individuals with Autism Spectrum Disorder (ASD): Implications for Assisting ASD Screening and Diagnosis. J. Autism Dev. Disord.

[65] De Vries L., Fouquaet I., Boets B., Naulaers G., Steyaert J. (2021). Autism spectrum disorder and pupillometry: A systematic review and meta-analysis. Neurosci. Biobehav. Rev..

[66] Tessier M.P., Pennestri M.H., Godbout R. (2018). Heart rate variability of typically developing and autistic children and adults before, during and after sleep. Int. J. Psychophysiol. Off. J. Int. Organ. Psychophysiol..

[67] Burr R.L. (2007). Interpretation of normalized spectral heart rate variability indices in sleep research: A critical review. Sleep.

[68] Davis E.A., Zhou W., Dailey M.J. (2018). Evidence for a direct effect of the autonomic nervous system on intestinal epithelial stem cell proliferation. Physiol. Rep..

[69] Busch R.A., Heneghan A.F., Pierre J.F., Wang X., Kudsk K.A. (2014). The enteric nervous system neuropeptide, bombesin, reverses innate immune impairments during parenteral nutrition. Ann. Surg..

[70] Bevins C.L., Salzman N.H. (2011). Paneth cells, antimicrobial peptides and maintenance of intestinal homeostasis. Nat. Rev. Microbiol..

[71] Heneghan A.F., Pierre J.F., Tandee K., Shanmuganayagam D., Wang X., Reed J.D., Steele J.L., Kudsk K.A. (2014). Parenteral nutrition decreases paneth cell function and intestinal bactericidal activity while increasing susceptibility to bacterial enteroinvasion. JPEN J. Parenter. Enter. Nutr..

[72] Ayabe T., Wulff H., Darmoul D., Cahalan M.D., Chandy K.G., Ouellette A.J. (2002). Modulation of mouse Paneth cell alpha-defensin secretion by mIKCa1, a Ca2+-activated, intermediate conductance potassium channel. J. Biol. Chem..

[73] Satoh Y., Ishikawa K., Oomori Y., Takeda S., Ono K. (1992). Bethanechol and a G-protein activator, NaF/AlCl3, induce secretory response in Paneth cells of mouse intestine. Cell Tissue Res..

[74] Satoh Y., Habara Y., Ono K., Kanno T. (1995). Carbamylcholine- and catecholamine-induced intracellular calcium dynamics of epithelial cells in mouse ileal crypts. Gastroenterology.

[75] Berridge M.J. (1995). Inositol trisphosphate and calcium signaling. Ann. N. Y. Acad. Sci. USA.

[76] Mathias A., Pais B., Favre L., Benyacoub J., Corthesy B. (2014). Role of secretory IgA in the mucosal sensing of commensal bacteria. Gut Microbes.

[77] Kaetzel C.S. (2014). Cooperativity among secretory IgA, the polymeric immunoglobulin receptor, and the gut microbiota promotes host-microbial mutualism. Immunol. Lett..

[78] Meyer-Hoffert U., Hornef M.W., Henriques-Normark B., Axelsson L.G., Midtvedt T., Putsep K., Andersson M. (2008). Secreted enteric antimicrobial activity localises to the mucus surface layer. Gut.

[79] Petnicki-Ocwieja T., Hrncir T., Liu Y.J., Biswas A., Hudcovic T., Tlaskalova-Hogenova H., Kobayashi K.S. (2009). Nod2 is required for the regulation of commensal microbiota in the intestine. Proc. Natl. Acad. Sci. USA.

[80] Salzman N.H., Hung K., Haribhai D., Chu H., Karlsson-Sjoberg J., Amir E., Teggatz P., Barman M., Hayward M., Eastwood D. (2010). Enteric defensins are essential regulators of intestinal microbial ecology. Nat. Immunol..

[81] Salzman N.H., Ghosh D., Huttner K.M., Paterson Y., Bevins C.L. (2003). Protection against enteric salmonellosis in transgenic mice expressing a human intestinal defensin. Nature.

[82] Cash H.L., Whitham C.V., Behrendt C.L., Hooper L.V. (2006). Symbiotic bacteria direct expression of an intestinal bactericidal lectin. Science.

[83] Mukherjee S., Vaishnava S., Hooper L.V. (2008). Multi-layered regulation of intestinal antimicrobial defense. Cell. Mol. Life Sci. CMLS.

[84] Sun X., Jobin C. (2014). Nucleotide-binding oligomerization domain-containing protein 2 controls host response to Campylobacter jejuni in Il10-/- mice. J. Infect. Dis..

[85] Benarroch E.E. (2019). Autonomic nervous system and neuroimmune interactions: New insights and clinical implications. Neurology.

[86] Furness J.B. (2012). The enteric nervous system and neurogastroenterology. Nat. Rev. Gastroenterol. Hepatol..

[87] Yamakawa K., Rajendran P.S., Takamiya T., Yagishita D., So E.L., Mahajan A., Shivkumar K., Vaseghi M. (2015). Vagal nerve stimulation activates vagal afferent fibers that reduce cardiac efferent parasympathetic effects. Am. J. Physiol. Heart Circ. Physiol..

[88] Saulnier D.M., Ringel Y., Heyman M.B., Foster J.A., Bercik P., Shulman R.J., Versalovic J., Verdu E.F., Dinan T.G., Hecht G. (2013). The intestinal microbiome, probiotics and prebiotics in neurogastroenterology. Gut Microbes.

[89] Derrien M., van Hylckama Vlieg J.E. (2015). Fate, activity, and impact of ingested bacteria within the human gut microbiota. Trends Microbiol..

[90] O’Hara A.M., Shanahan F. (2006). The gut flora as a forgotten organ. EMBO Rep..

[91] Furness J.B. (2006). Novel gut afferents: Intrinsic afferent neurons and intestinofugal neurons. Auton. Neurosci..

[92] Chesne J., Cardoso V., Veiga-Fernandes H. (2019). Neuro-immune regulation of mucosal physiology. Mucosal. Immunol..

[93] De Jonge W.J. (2013). The Gut’s Little Brain in Control of Intestinal Immunity. ISRN Gastroenterol..

[94] Bellono N.W., Bayrer J.R., Leitch D.B., Castro J., Zhang C., O’Donnell T.A., Brierley S.M., Ingraham H.A., Julius D. (2017). Enterochromaffin Cells Are Gut Chemosensors that Couple to Sensory Neural Pathways. Cell.

[95] Campos-Rodriguez R., Godinez-Victoria M., Abarca-Rojano E., Pacheco-Yepez J., Reyna-Garfias H., Barbosa-Cabrera R.E., Drago-Serrano M.E. (2013). Stress modulates intestinal secretory immunoglobulin A. Front. Integr. Neurosci..

[96] Abot A., Cani P.D., Knauf C. (2018). Impact of Intestinal Peptides on the Enteric Nervous System: Novel Approaches to Control Glucose Metabolism and Food Intake. Front. Endocrinol..

[97] Lach G., Schellekens H., Dinan T.G., Cryan J.F. (2018). Anxiety, Depression, and the Microbiome: A Role for Gut Peptides. Neurotherapeutics.

[98] McVey Neufeld K.A., Perez-Burgos A., Mao Y.K., Bienenstock J., Kunze W.A. (2015). The gut microbiome restores intrinsic and extrinsic nerve function in germ-free mice accompanied by changes in calbindin. Neurogastroenterol. Motil..

[99] McVey Neufeld K.A., Mao Y.K., Bienenstock J., Foster J.A., Kunze W.A. (2013). The microbiome is essential for normal gut intrinsic primary afferent neuron excitability in the mouse. Neurogastroenterol. Motil..

[100] Bufe B., Schumann T., Kappl R., Bogeski I., Kummerow C., Podgorska M., Smola S., Hoth M., Zufall F. (2015). Recognition of bacterial signal peptides by mammalian formyl peptide receptors: A new mechanism for sensing pathogens. J. Biol. Chem..

[101] Bufe B., Zufall F. (2016). The sensing of bacteria: Emerging principles for the detection of signal sequences by formyl peptide receptors. Biomol. Concepts.

[102] Cianciulli A., Acquafredda A., Cavallo P., Saponaro C., Calvello R., Mitolo V., Panaro M.A. (2009). f-Met-Leu-Phe stimulates nitric oxide production in chick embryo neurons: The role of NF-kB. Immunopharmacol. Immunotoxicol..

[103] Monstein H.J., Grahn N., Truedsson M., Ohlsson B. (2004). Oxytocin and oxytocin-receptor mRNA expression in the human gastrointestinal tract: A polymerase chain reaction study. Regul. Pept..

[104] Ohlsson B., Forsling M.L., Rehfeld J.F., Sjolund K. (2002). Cholecystokinin stimulation leads to increased oxytocin secretion in women. Eur. J. Surg..

[105] Ohlsson B., Truedsson M., Djerf P., Sundler F. (2006). Oxytocin is expressed throughout the human gastrointestinal tract. Regul. Pept..

[106] Petersson M., Alster P., Lundeberg T., Uvnas-Moberg K. (1996). Oxytocin increases nociceptive thresholds in a long-term perspective in female and male rats. Neurosci. Lett..

[107] Uvnas-Moberg K., Arn I., Theorell T., Jonsson C.O. (1991). Gastrin, somatostatin and oxytocin levels in patients with functional disorders of the gastrointestinal tract and their response to feeding and interaction. J. Psychosom. Res..

[108] Alfven G. (2004). Plasma oxytocin in children with recurrent abdominal pain. J. Pediatr. Gastroenterol. Nutr..

[109] Welch M.G., Tamir H., Gross K.J., Chen J., Anwar M., Gershon M.D. (2009). Expression and developmental regulation of oxytocin (OT) and oxytocin receptors (OTR) in the enteric nervous system (ENS) and intestinal epithelium. J. Comp. Neurol..

[110] Desbonnet L., Clarke G., Traplin A., O’Sullivan O., Crispie F., Moloney R.D., Cotter P.D., Dinan T.G., Cryan J.F. (2015). Gut microbiota depletion from early adolescence in mice: Implications for brain and behaviour. Brain. Behav. Immun..

[111] Desbonnet L., Clarke G., Shanahan F., Dinan T.G., Cryan J.F. (2014). Microbiota is essential for social development in the mouse. Mol. Psychiatry.

[112] Sternberg E.M. (2006). Neural regulation of innate immunity: A coordinated nonspecific host response to pathogens. Nat. Rev. Immunol..

[113] Wostmann B.S. (1981). The germfree animal in nutritional studies. Annu. Rev. Nutr..

[114] Hooper L.V., Gordon J.I. (2001). Commensal host-bacterial relationships in the gut. Science.

[115] Shroff K.E., Meslin K., Cebra J.J. (1995). Commensal enteric bacteria engender a self-limiting humoral mucosal immune response while permanently colonizing the gut. Infect. Immun..

[116] Furness J.B., Kunze W.A., Bertrand P.P., Clerc N., Bornstein J.C. (1998). Intrinsic primary afferent neurons of the intestine. Prog. Neurobiol..

[117] Mao Y.K., Kasper D.L., Wang B., Forsythe P., Bienenstock J., Kunze W.A. (2013). Bacteroides fragilis polysaccharide A is necessary and sufficient for acute activation of intestinal sensory neurons. Nat. Commun..

[118] Collins J., Borojevic R., Verdu E.F., Huizinga J.D., Ratcliffe E.M. (2014). Intestinal microbiota influence the early postnatal development of the enteric nervous system. Neurogastroenterol. Motil..

[119] Straub R.H., Wiest R., Strauch U.G., Harle P., Scholmerich J. (2006). The role of the sympathetic nervous system in intestinal inflammation. Gut.

[120] Hart A., Kamm M.A. (2002). Review article: Mechanisms of initiation and perpetuation of gut inflammation by stress. Aliment. Pharm..

[121] Savidge T.C., Newman P., Pothoulakis C., Ruhl A., Neunlist M., Bourreille A., Hurst R., Sofroniew M.V. (2007). Enteric glia regulate intestinal barrier function and inflammation via release of S-nitrosoglutathione. Gastroenterology.

[122] Jarillo-Luna A., Rivera-Aguilar V., Martinez-Carrillo B.E., Barbosa-Cabrera E., Garfias H.R., Campos-Rodriguez R. (2008). Effect of restraint stress on the population of intestinal intraepithelial lymphocytes in mice. Brain Behav. Immun..

[123] Martinez-Carrillo B.E., Godinez-Victoria M., Jarillo-Luna A., Oros-Pantoja R., Abarca-Rojano E., Rivera-Aguilar V., Yepez J.P., Sanchez-Torres L.E., Campos-Rodriguez R. (2011). Repeated restraint stress reduces the number of IgA-producing cells in Peyer’s patches. Neuroimmunomodulation.

[124] Reyna-Garfias H., Miliar A., Jarillo-Luna A., Rivera-Aguilar V., Pacheco-Yepez J., Baeza I., Campos-Rodriguez R. (2010). Repeated restraint stress increases IgA concentration in rat small intestine. Brain Behav. Immun..

[125] Mantis N.J., Rol N., Corthesy B. (2011). Secretory IgA’s complex roles in immunity and mucosal homeostasis in the gut. Mucosal. Immunol..

[126] Mayer E.A., Tillisch K., Gupta A. (2015). Gut/brain axis and the microbiota. J. Clin. Investig..

[127] Bercik P., Park A.J., Sinclair D., Khoshdel A., Lu J., Huang X., Deng Y., Blennerhassett P.A., Fahnestock M., Moine D. (2011). The anxiolytic effect of Bifidobacterium longum NCC3001 involves vagal pathways for gut-brain communication. Neurogastroenterol. Motil..

[128] Maiti A.K., Sharba S., Navabi N., Linden S.K. (2018). Colonic levels of vasoactive intestinal peptide decrease during infection and exogenous VIP protects epithelial mitochondria against the negative effects of IFNgamma and TNFalpha induced during Citrobacter rodentium infection. PLoS ONE.

[129] Coquenlorge S., Duchalais E., Chevalier J., Cossais F., Rolli-Derkinderen M., Neunlist M. (2014). Modulation of lipopolysaccharide-induced neuronal response by activation of the enteric nervous system. J. Neuroinflamm..

[130] Saurer T.B., Ijames S.G., Lysle D.T. (2006). Neuropeptide Y Y1 receptors mediate morphine-induced reductions of natural killer cell activity. J. Neuroimmunol..

[131] Bonaz B., Sinniger V., Pellissier S. (2016). Anti-inflammatory properties of the vagus nerve: Potential therapeutic implications of vagus nerve stimulation. J. Physiol.

[132] Rosas-Ballina M., Olofsson P.S., Ochani M., Valdes-Ferrer S.I., Levine Y.A., Reardon C., Tusche M.W., Pavlov V.A., Andersson U., Chavan S. (2011). Acetylcholine-synthesizing T cells relay neural signals in a vagus nerve circuit. Science.

[133] Reardon C., Duncan G.S., Brustle A., Brenner D., Tusche M.W., Olofsson P.S., Rosas-Ballina M., Tracey K.J., Mak T.W. (2013). Lymphocyte-derived ACh regulates local innate but not adaptive immunity. Proc. Natl. Acad. Sci. USA.

[134] Wang H., Yu M., Ochani M., Amella C.A., Tanovic M., Susarla S., Li J.H., Wang H., Yang H., Ulloa L. (2003). Nicotinic acetylcholine receptor alpha7 subunit is an essential regulator of inflammation. Nature.

[135] Miller W.L. (2018). The Hypothalamic-Pituitary-Adrenal Axis: A Brief History. Horm. Res. Paediatr..

[136] Cailotto C., Gomez-Pinilla P.J., Costes L.M., van der Vliet J., Di Giovangiulio M., Nemethova A., Matteoli G., Boeckxstaens G.E. (2014). Neuro-anatomical evidence indicating indirect modulation of macrophages by vagal efferents in the intestine but not in the spleen. PLoS ONE.

[137] Birrenbach T., Bocker U. (2004). Inflammatory bowel disease and smoking: A review of epidemiology, pathophysiology, and therapeutic implications. Inflamm. Bowel. Dis..

[138] Rosas-Ballina M., Ochani M., Parrish W.R., Ochani K., Harris Y.T., Huston J.M., Chavan S., Tracey K.J. (2008). Splenic nerve is required for cholinergic antiinflammatory pathway control of TNF in endotoxemia. Proc. Natl. Acad. Sci. USA.

[139] Engelstoft M.S., Egerod K.L., Holst B., Schwartz T.W. (2008). A gut feeling for obesity: 7TM sensors on enteroendocrine cells. Cell Metab..

[140] Thomas C., Gioiello A., Noriega L., Strehle A., Oury J., Rizzo G., Macchiarulo A., Yamamoto H., Mataki C., Pruzanski M. (2009). TGR5-mediated bile acid sensing controls glucose homeostasis. Cell Metab..

[141] Brubaker P.L., Anini Y. (2003). Direct and indirect mechanisms regulating secretion of glucagon-like peptide-1 and glucagon-like peptide-2. Can. J. Physiol. Pharm..

[142] Sandoval D., Dunki-Jacobs A., Sorrell J., Seeley R.J., D’Alessio D.D. (2013). Impact of intestinal electrical stimulation on nutrient-induced GLP-1 secretion in vivo. Neurogastroenterol. Motil..

[143] Holst J.J. (2007). The physiology of glucagon-like peptide 1. Physiol. Rev..

[144] Cani P.D., Possemiers S., Van de Wiele T., Guiot Y., Everard A., Rottier O., Geurts L., Naslain D., Neyrinck A., Lambert D.M. (2009). Changes in gut microbiota control inflammation in obese mice through a mechanism involving GLP-2-driven improvement of gut permeability. Gut.

[145] Hsieh J., Longuet C., Maida A., Bahrami J., Xu E., Baker C.L., Brubaker P.L., Drucker D.J., Adeli K. (2009). Glucagon-like peptide-2 increases intestinal lipid absorption and chylomicron production via CD36. Gastroenterology.

[146] Rowland K.J., Brubaker P.L. (2011). The "cryptic" mechanism of action of glucagon-like peptide-2. Am. J. Physiol. Gastrointest. Liver Physiol..

[147] Buchman A.L., Katz S., Fang J.C., Bernstein C.N., Abou-Assi S.G., Teduglutide Study G. (2010). Teduglutide, a novel mucosally active analog of glucagon-like peptide-2 (GLP-2) for the treatment of moderate to severe Crohn’s disease. Inflamm. Bowel. Dis..

[148] Jeppesen P.B., Gilroy R., Pertkiewicz M., Allard J.P., Messing B., O’Keefe S.J. (2011). Randomised placebo-controlled trial of teduglutide in reducing parenteral nutrition and/or intravenous fluid requirements in patients with short bowel syndrome. Gut.

[149] Hyland N.P., Sjoberg F., Tough I.R., Herzog H., Cox H.M. (2003). Functional consequences of neuropeptide Y Y 2 receptor knockout and Y2 antagonism in mouse and human colonic tissues. Br. J. Pharm..

[150] Cox H.M. (2007). Neuropeptide Y receptors; antisecretory control of intestinal epithelial function. Auton. Neurosci..

[151] Stengel A., Goebel M., Wang L., Tache Y., Sachs G., Lambrecht N.W. (2010). Differential distribution of ghrelin-O-acyltransferase (GOAT) immunoreactive cells in the mouse and rat gastric oxyntic mucosa. Biochem. Biophys. Res. Commun..

[152] Cho H.J., Robinson E.S., Rivera L.R., McMillan P.J., Testro A., Nikfarjam M., Bravo D.M., Furness J.B. (2014). Glucagon-like peptide 1 and peptide YY are in separate storage organelles in enteroendocrine cells. Cell Tissue Res..

[153] Young R.L. (2011). Sensing via intestinal sweet taste pathways. Front. Neurosci..

[154] Sigalet D.L., Wallace L.E., Holst J.J., Martin G.R., Kaji T., Tanaka H., Sharkey K.A. (2007). Enteric neural pathways mediate the anti-inflammatory actions of glucagon-like peptide 2. Am. J. Physiol Gastrointest. Liver Physiol..

[155] Shirazi-Beechey S.P., Moran A.W., Batchelor D.J., Daly K., Al-Rammahi M. (2011). Glucose sensing and signalling; regulation of intestinal glucose transport. Proc. Nutr Soc..

[156] Lundgren O. (2002). Enteric nerves and diarrhoea. Pharm. Toxicol..

[157] Gwynne R.M., Ellis M., Sjovall H., Bornstein J.C. (2009). Cholera toxin induces sustained hyperexcitability in submucosal secretomotor neurons in guinea pig jejunum. Gastroenterology.

[158] Tropini C., Moss E.L., Merrill B.D., Ng K.M., Higginbottom S.K., Casavant E.P., Gonzalez C.G., Fremin B., Bouley D.M., Elias J.E. (2018). Transient Osmotic Perturbation Causes Long-Term Alteration to the Gut Microbiota. Cell.

[159] Darkoh C., Plants-Paris K., Bishoff D., DuPont H.L. (2019). Clostridium difficile Modulates the Gut Microbiota by Inducing the Production of Indole, an Interkingdom Signaling and Antimicrobial Molecule. mSystems.

